# A novel echocardiographic estimate of pulmonary vascular resistance employing the hydraulic analogy to Ohm’s law^☆^

**DOI:** 10.1016/j.ijcha.2022.101121

**Published:** 2022-09-09

**Authors:** Ashwin Venkateshvaran, Erik Tossavainen, Charlie Borneteg, Hande Oktay Tureli, Davide Vanoli, Lars H. Lund, Frank Flachskampf, Per Lindqvist

**Affiliations:** a Department of Medicine, Cardiology Unit, Karolinska Institutet, Stockholm, Sweden; b Department of Cardiology, Public Health & Clinical Medicine, Umeå University, Umeå, Sweden; c Department of Clinical Physiology, Surgical & Perioperative Sciences, Umeå University, Umeå, Sweden; d Department of Medical Sciences, Uppsala University, and Clinical Physiology and Cardiology, Uppsala University Clinic, Uppsala, Sweden

**Keywords:** Doppler echocardiography, Right heart catheterization, Pulmonary hypertension, Heart failure

## Abstract

**Background:**

Assessment of pulmonary vascular resistance (PVR) is critical for accurate diagnosis and optimal pharmacotherapy in pulmonary hypertension. We aimed to test the diagnostic performance of a novel, Doppler-based method to evaluate PVR based on Ohm’s law (PVR_echo_) using pragmatic estimates of pulmonary capillary wedge pressure (PCWP).

**Methods and results:**

Simultaneous right heart catheterization (RHC) and echocardiography was performed in a derivation cohort of 111 patients in sinus rhythm referred for PH evaluation and PVR_echo_ independently validated in 238 patients. PVR_echo_ was calculated using pulmonary artery mean pressure estimates (PAMP_echo_) obtained from peak tricuspid gradient employing a fixed right atrial pressure estimate, PCWP_echo_ was estimated as 10 or 20 mmHg using age-related mitral E/A cut-offs and cardiac output from left ventricular outflow. In the derivation cohort, both PAMP_echo_ and PCWP_echo_ estimates demonstrated excellent agreement with catheterization measurements. PVR_echo_ was highly feasible, demonstrated negligible bias and excellent agreement with PVR_RHC_ (Bias = −0.58, SD 2.2 mmHg) and outperformed the Abbas method to identify PVR_RHC_ > 3WU (AUC = 0.85 vs. 0.70; p = 0.02). In the validation cohort, PVR_echo_ preserved good invasive agreement with negligible bias, displayed strong diagnostic performance (AUC = 0.84) and significant ability to distinguish isolated post-capillary from combined post- and pre-capillary pulmonary hypertension (PH) subgroups (AUC = 0.77).

**Conclusion:**

PVR_echo_ based on Ohm’s law employing pragmatic estimates of PCWP_echo_ demonstrates excellent agreement with invasive reference standard measurements and strong diagnostic ability to identify elevated PVR_RHC_. This novel approach may be useful during therapy selection to distinguish PH hemodynamic subgroups.

## Graphical abstract

**Figure:**
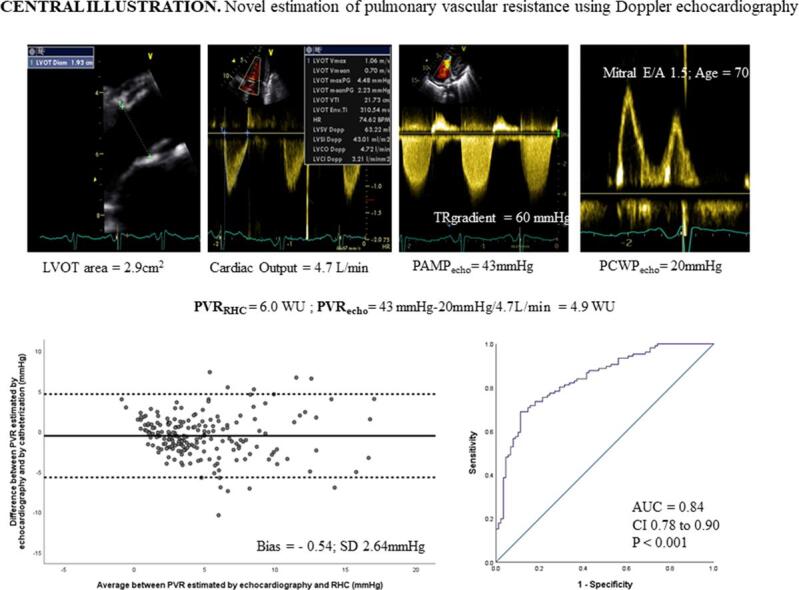

## 1 Background

Pulmonary hypertension (PH) is a chronic, progressive disease common in multiple clinical disorders and associated with poor long-term outcomes. Hemodynamic classification of patients with PH necessitates estimation of pulmonary vascular resistance (PVR), a static index of impedance that reflects pathological remodeling of the distal arterioles and alterations to the pulmonary vascular bed. Accurate quantification of PVR is important for a number of reasons. As a hemodynamic diagnostic indicator, PVR is integral to classifying PH subjects as having isolated post-capillary or combined post- and pre-capillary PH. [1] Further, PVR is an independent risk factor in the setting of heart failure (HF) and a strong predictor for reduced exercise capacity. [2] In multiple randomized clinical trials, reduction in PVR is associated with improvements of traditional risk stratification indices such as 6-minute walk test, WHO functional class and NT-proBNP.[3], [4]

Reference-standard PVR is assessed using invasive right heart catheterization (RHC). Doppler-based approaches have been proposed [5], [6], [7], [8], [9], and present distinct advantages of being non-invasive, low-cost and highly accessible. However, their accuracy has been debated [10] and clinical utility may be limited by method complexity.[9] We have previously presented a novel, Doppler-based approach to assess PVR in a pre-capillary PH cohort based on the hydraulic analogy to Ohm’s relationship. In that study, we employed a fixed, non-elevated PCWP estimate in all patients considering their pre-capillary PH status.[11] In the current study, we hypothesized that incorporation of a reliable, clinically relevant and simplified estimate of PCWP would allow wider application of this approach to the general PH population. We aimed to evaluate the accuracy of a Doppler-derived algorithm based on Ohm’s law to evaluate PVR using routinely-assessed echocardiographic variables in a general population of symptomatic patients referred for PH evaluation.

## 2 Methods

### 2.1 Study population

Consecutive patients with unexplained breathlessness referred for clinically-indicated RHC to Norrlands University Hospital between 2010 and 2015 were retrospectively analyzed. Patients with intracardiac or extracardiac shunts and severe valvular disorders were excluded prior to enrollment. Patients with atrial fibrillation or significant arrhythmia and no tricuspid regurgitation (TR) signals on echocardiography were excluded from the final cohort. Ethics committee approval was obtained prior to study enrollment (DNR 07–092M) and all patients provided written informed consent.

### 2.2 Right heart catheterization

RHC was performed by experienced operators blinded to echocardiographic data. Venous access was obtained by inserting an introducer in a medial cubital vein or in the femoral vein. A retrograde, right-heart catheterization was then performed using a Swan-Ganz pulmonary artery catheter (Edwards Lifesciences). Mean right atrial pressure (RAP), systolic and right ventricular end-diastolic pressures, pulmonary artery systolic, mean and diastolic pressures (PASP_RHC_, PAMP_RHC_ and PADP_RHC_ respectively), and mean pulmonary capillary wedge pressure (PCWP_RHC_) were measured. Blood samples for estimation of oxygen saturation were drawn from the superior and inferior vena cava, as well as right atrium, and samples from the pulmonary and femoral arteries were used for screening for intra-cardiac shunts. Cardiac output (CO_RHC_) was determined by thermodilution. Pulmonary vascular resistance was calculated using the equation PAMP_RHC_ − PCWP_RHC_ (*trans*-pulmonary gradient) divided by CO_RHC_.

### 2.3 Echocardiography

Doppler Echocardiographic examination was performed by an experienced echocardiographer (PL) with > 15 years’ experience on-table, during RHC using a Vivid 7 system (GE Ultrasound, Horten, Norway) equipped with an adult 1.5–4.3 MHz phased array transducer. Standard views from the parasternal long and short axis and apical views were used in keeping with current recommendations.[12] Gray-scale images were obtained at 50 – 80 frames/sec and Doppler acquisitions at a sweep speed of 100 mm/sec. PASP using echocardiography (PASP_echo_) was estimated using Continuous-Wave (CW) Doppler from the tricuspid regurgitation (TR) jet considering the most optimal of signals across multiple acoustic windows. Stroke volume (SV) was measured using Pulse-Wave (PW) Doppler at the level of the LV outflow tract, and CO_echo_ calculated by multiplying SV with heart rate. Mitral flow interrogation was performed in the 4-chamber view with the PW sample-volume placed between the mitral leaflets tips and measurements taken at end expiration. Early transmitral (E) and late diastolic (A) velocities were obtained after optimal sample alignment and E/A ratio was subsequently computed. Off-line analysis was performed using a commercially available software system (General Electric, EchoPAC PC version 11.0.0, GE Ultrasound, Waukesha, Wisconsin). Mean of three consecutive tracings were used to estimate a representative measurement.

**Fig. 1:**
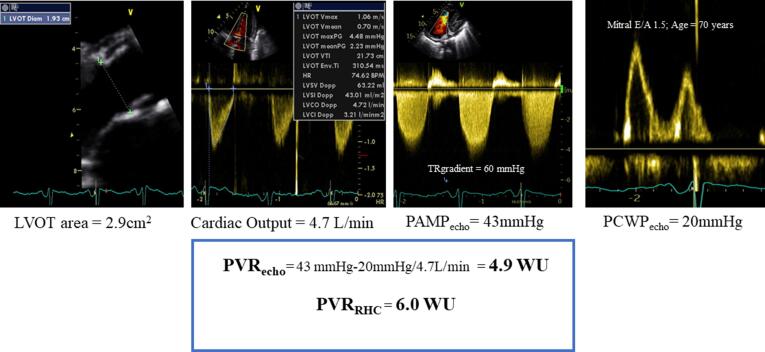
Illustration of PVR assessment using routinely acquired variables employing the Ohm’s relationship (PVR_echo_) and corresponding PVR obtained using right heart catheterization (PVR_RHC_).

Assessment of PVR using echocardiography (PVR_echo_) was estimated using the hydraulic analogy to the Ohm’s relationship, i.e., PVR = (PAMP − PCWP)/CO employing echocardiographic surrogates for each of the variables employed in conventional equation, i.e transpulmonary gradient and ventricular output_._ PAMP_echo_ was calculated using the formula PASP_echo_ × 0.61 + 2 mmHg according to Chemla et al. [13] PASP_echo_ was estimated employing the peak *trans*-tricuspid retrograde pressure drop adding a fixed right atrial pressure (RAP) of 7 mmHg. [14] Additional analysis was performed to estimate PASP_echo_ employing current recommendations considering inferior vena cava size and respiratory dynamics.[12] PCWP_echo_ was estimated based on combination of interpretation of Mitral E/A ratio and age. PCWP_echo_ was assigned a simplified estimate of 20 mmHg in younger patients (<50 years) if E/A ratio was > 2, and older patients (≥50 years) if E/A was > 1.4. In all other cases PCWP_echo_ was estimated as 10 mmHg. An illustration of PVR_echo_ assessment employing this novel approach has been provided in Fig. 1**.**

### 2.4 Statistical analysis

Continuous variables were expressed as mean ± SD for parametric variables or median (interquartile range) for non-parametric variables. Categorical variables were expressed as numbers and percentage. PAMP_echo_ and PCWP_echo_ were computed as described earlier. Correlations between reference standard invasive measurements and novel echocardiographic estimates were tested using Pearson's 2-tailed test. Inter-technique agreement between echocardiographic and invasive measurements was tested using Bland-Altman analysis and calculated ĸ coefficients, where 0 to 0.2 was judged as slight; 0.21 to 0.4 as fair; 0.41 to 0.6 as moderate; 0.61 to 0.80 as good and > 0.8 as excellent. Receiver operating characteristics (ROC) analysis was performed to evaluate the diagnostic performance of PVR_echo_ to identify PVR_RHC_ > 3WU. Delong’s method was used to compare area under the curve using the novel PVR_echo_ algorithm and conventional echocardiographic assessment. Sensitivity and specificity were calculated. IBM SPSS statistics version 23.0 was employed for analysis. A p-value < 0.05 was considered statistically significant.

## 3 Results

Of 145 patients referred for RHC in the derivation cohort, 32 patients with AF or significant arrythmia and 2 with no TR signals were excluded. In effect, 111 (mean age 61 ± 14 years; 36 male) with sinus rhythm were included in the analysis. In the analyzed patient cohort, trivial or mild TR was seen in 88 patients (79%), moderate in 20 (18%) and moderate to severe in 3 (3%). No patients demonstrated free-flowing severe/torrential TR. On catheterization, 35 (32%) did not demonstrate PH and 76 (68%) had PH in keeping with the revised hemodynamic definition of PAMP_RHC_ > 20 mmHg at rest. [15] Fifty-one patients (46%) demonstrated elevated invasive PVR_RHC_ (>3 WU). When PH patients were classified by etiology, 36 (32%) demonstrated pulmonary arterial hypertension, 40 (36%) had PH secondary to left heart disease, 9 (8%) had PH due to lung disease, 11(10%) demonstrated chronic thromboembolic PH, and 15 (14%) demonstrated PH due to multifactorial mechanisms. When classified by hemodynamic status, 46 PH patients (61%) demonstrated pre-capillary PH (PCWP ≤ 15 mmHg) and 30 (39%) demonstrated post-capillary PH (PCWP > 15 mmHg). Among post-capillary PH patients, 18 (60%) demonstrated isolated post-capillary PH (PCWP > 15 mmHg and PVR ≤ 3WU) and 12 (40%) demonstrated combined post- and pre-capillary PH (PCWP > 15 mmHg and PVR > 3WU).

Baseline characteristics of the derivation cohort are presented in Table 1, stratified by PVR_RHC_ subgroups. Patients with elevated PVR_RHC_ demonstrated significantly smaller LV volumes and higher EF, larger right atrial (RA) and RV size, and lower RV longitudinal function seen both in lower TAPSE and RV free wall strain (p < 0.05 for all group comparisons).

**Table 1:** Clinical Characteristics, right heart catheterization and echocardiographic data of patient population in the derivation cohort, grouped by PVR subgroups. Data presented as mean ± SD/ median (Q1; Q3) or number (%).

	**All** **(n = 111)**	**PVR ≤ 3WU** **(n = 60; 54%)**	**PVR > 3WU** **(n = 51; 46%)**	**P-value**
**Clinical Characteristics**				
Age (years)	61 ± 14	59 ± 15	63 ± 13	0.15
Female	75 (68)	42 (70)	33 (65)	0.44
Diabetes	13 (12)	10 (17)	3 (6)	0.68
Hypertension	40 (36)	25 (42)	15 (29)	0.23
Ischaemic heart disease	14 (13)	7 (12)	7 (14)	0.13
Heart rate (bpm)	74 ± 14	72 ± 15	76 ± 13	0.10
Body surface area (m^2^)	1.86 ± 0.25	1.89 ± 0.27	1.83 ± 0.21	0.30
Systolic blood pressure (mmHg)	132 ± 20	132 ± 19	133 ± 20	0.95
Diastolic blood pressure (mmHg)	77 ± 9	75 ± 8	79 ± 10	0.01
NTproBNP (ng/L)	477 (181;1582)	341 (152;1375)	668 (267;1933)	0.20

**Right heart catheterization**				
RAP_mean_ (mmHg)	7 ± 5	7 ± 4	8 ± 6	0.08
PAP_mean_ (mmHg)	32 ± 15	24 ± 9	43 ± 15	<0.001
PCWP (mmHg)	12 ± 6	13 ± 7	11 ± 5	0.05
TPG (mmHg)	20 ± 14	11 ± 5	30 ± 14	<0.001
PVR (WU)	4.2 ± 3.4	2.0 ± 0.7	6.8 ± 3.3	<0.001
Cardiac output (L/min)	5.3 ± 1.6	5.7 ± 1.9	4.9 ± 1.2	0.01

**Echocardiography**				
LV end-diastolic volume (ml)	87 ± 50	101 ± 56	72 ± 38	0.003
LV end-systolic volume (ml)	43 ± 38	53 ± 46	32 ± 22	0.005
LVEF (%)	54 ± 13	51 ± 13	57 ± 12	0.03
RV basal diameter (mm)	41 ± 8	39 ± 9	44 ± 6	0.002
RA area (cm^2^)	19 ± 7	18 ± 7	21 ± 6	0.04
TAPSE (mm)	20 ± 5	21 ± 5	18 ± 4	0.01
RV SL (%)	17 ± 7	19 ± 7	15 ± 6	0.005
Mitral E wave (m/s)	73 ± 27	81 ± 22	65 ± 29	0.001
Mitral E/A ratio	1.3 ± 0.8	1.5 ± 0.9	1.1 ± 0.6	0.01
Mitral E/e’_mean_	10 ± 5	10 ± 5	10 ± 5	0.41
TR peak velocity (m/s)	3.4 ± 0.7	2.7 ± 0.3	3.7 ± 0.6	<0.001
RVSP (mmHg)	56 ± 22	43 ± 16	67 ± 21	<0.001

NTproBNP, N-terminal pro-B-type natriuretic peptide; RAP, right atrial pressure; PAP, pulmonary artery pressure; PCWP, pulmonary capillary wedge pressure; TPG, transpulmonary gradient; PVR, pulmonary vascular resistance; LV, left ventricle; EF, ejection fraction; RV, right ventricular; TAPSE, tricuspid annular plane systolic excursion; TR, tricuspid regurgitation.

### 3.1 Feasibility and diagnostic accuracy of PAMP_echo_ to represent PAMP_RHC_

**Fig. 2:**
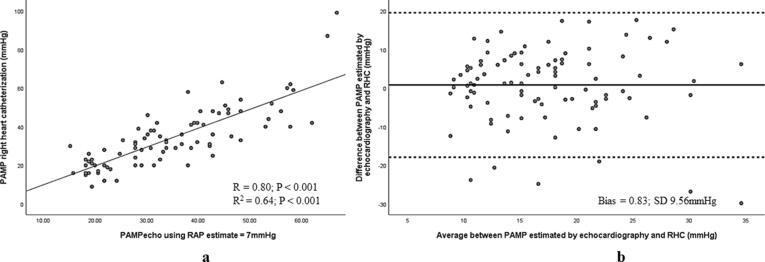
(a) Scatter plot demonstrating strong relationship between PAMP_echo_ PAMP_RHC_ and (b) Bland-Altman plot displaying negligible bias and good agreement between modalities in the derivation cohort.

TR velocity could be adequately assessed in 96 (86%), and echocardiographic estimates of RAP from inferior vena cava size and collapse in 92 (83%) of patients in the derivation cohort. Applying ASE/EACVI recommended estimates of RAP (12), PAMP_echo_ using the Chemla’s equation demonstrated strong correlation (r = 0.82, r^2^ = 0.67; p < 0.001 for both) and minimal bias (Bias = 0.66; SD 9.22 mmHg) with PAMP_RHC_. Employing a simplified approach using a fixed, mean RAP_echo_ (7 mmHg), strong correlation (r = 0.80, r^2^ = 0.64; p < 0.001 for both) **(**Fig. 2**a)** and excellent agreement with PAMP_RHC_ was preserved with a relatively higher spread of data points (Bias = 0.83; SD 9.56 mmHg) **(**Fig. 2**b).**

### 3.2 Diagnostic **accuracy of age-dependent mitral E/A to represent PCWP**_RHC_

Mitral E/A ratio was highly feasible (95%), demonstrated a strong positive correlation with PCWP_RHC_ (r = 0.65, p < 0.001) and outperformed other echocardiographic surrogates i.e., Mitral E (r = 0.43; p < 0.001), E/e’ (0.46; p < 0.001), TR velocity (r = 0.01; p = 0.90) and LA volume index (0.38; p < 0.001). Further, mitral E/A demonstrated excellent ability to identify elevated PCWP_RHC_ (AUC = 0.84; CI 0.73 to 0.94; p < 0.001) and E/A cut-off > 2 demonstrated 50% sensitivity and 100% specificity to identify elevated PCWP_RHC_ in the total cohort.

**Fig. 3:**
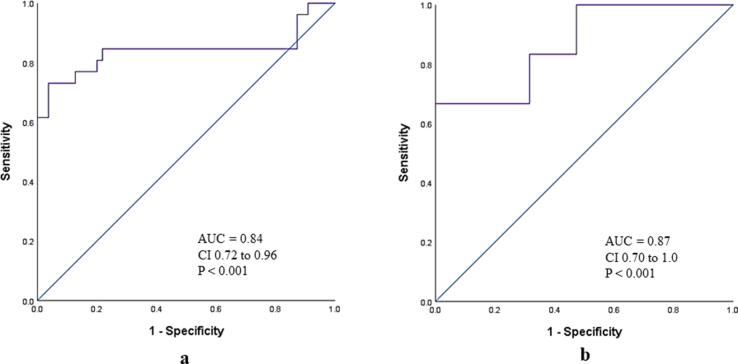
(a) ROC curve demonstrating diagnostic ability of mitral E/A > 1.4 to identify PCWP_RHC_ > 15 mm Hg in patients ≥ 50 years and (b) diagnostic ability of mitral E/A 2.0 to identify PCWP_RHC_ > 15 mmHg in patients < 50 years in the derivation cohort.

Eighty-four patients (76%) were ≥ 50 years and 27 (24%) were < 50 years old in the derivation cohort. In the older (≥50 years) sub-group, mitral E/A > 1.4 demonstrated 69% sensitivity, 96% specificity, 90% PPV, 87% NPV and 88% accuracy to identify elevated PCWP_RHC_ (AUC = 0.84, CI 0.72 to 0.96; p < 0.001) **(**Fig. 3**a)**. Lower sensitivity (46%) and accuracy (82%) but excellent specificity (100%) was observed when E/A > 2 was considered as cut-off in this subgroup. In the younger group (<50 years) mitral E/A cut-off > 2 demonstrated 67% sensitivity, 100% specificity, 100% PPV, 91% NPV and 92% accuracy (AUC = 0.87, CI 0.70 to 1.0) **(**Fig. 3**b)**.

**Fig. 4:**
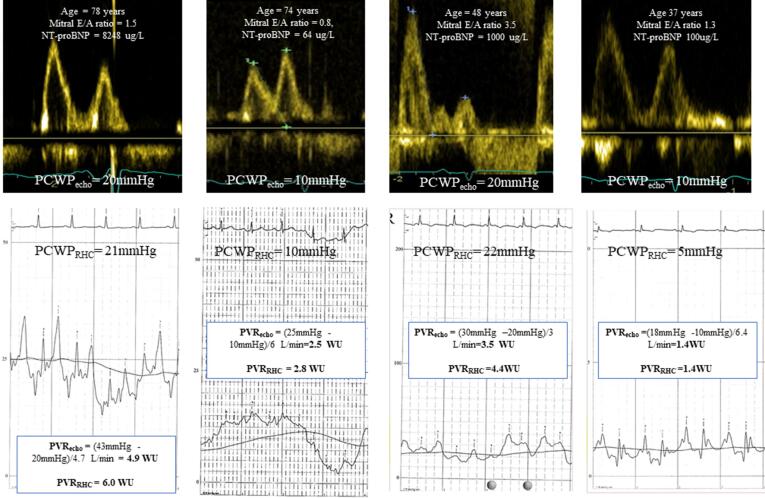
Illustration of PCWP_echo_ assessment based on age and mitral E/A ratio and corresponding invasive PCWP and PVR_echo._

Simplified estimation of PCWP_echo_ as being non-elevated (10 mmHg) or elevated (20 mmHg) considering age in addition to mitral E/A as described in our methods demonstrated excellent diagnostic ability to identify PCWP_RHC_ (AUC = 0.84; CI 0.70 to 0.94; p < 0.001) in addition to good agreement with PCWP_RHC_ (*Kappa* coefficient = 0.69). When compared with the current 2016 ASE/EACVI algorithm to determine elevated LV filling pressure, age-dependent mitral E/A demonstrated higher feasibility (95 vs 87%), specificity (97 vs 93%) PPV (91 vs. 32%) and modestly higher accuracy (89 vs 87%) **(**Table 3**)**. An illustration displaying age-dependent mitral E/A ratio and corresponding PCWP_RHC_ in addition to PVR_echo_ and corresponding PVR_RHC_ is provided in Fig. 4 .

**Table 2:** Clinical Characteristics, right heart catheterization and echocardiographic data of patient population in the validation cohort. Data presented as mean ± SD/ median (Q1; Q3) or number (%).

	**All Patients(n = 238)**
**Clinical Characteristics**	
Age (years)	58 ± 16
Female	120 (50)
Diabetes	27 (11)
Hypertension	103 (43)
Ischaemic heart disease	22 (9)
Heart rate (bpm)	72 ± 13
Body surface area (m^2^)	1.87 ± 0.24
Systolic blood pressure (mmHg)	121 ± 23
Diastolic blood pressure (mmHg)	68 ± 12
NTproBNP (ng/L)	1395 (349:2765)

**Right heart catheterization**	
RAP_mean_ (mmHg)	7 ± 5
PAP_mean_ (mmHg)	32 ± 13
PCWP (mmHg)	14 ± 7
TPG (mmHg)	19 ± 13
PVR (WU)	4.3 ± 3.5
Cardiac output (L/min)	5.3 ± 1.6

**Echocardiography**	
LV end-diastolic volume (ml)	114 ± 58
LV end-systolic volume (ml)	55 ± 53
LVEF (%)	55 ± 15
RV basal diameter (mm)	41 ± 8
RA area (cm^2^)	20 ± 7
TAPSE (mm)	17 ± 6
RV SL (%)	17 ± 8
Mitral E wave (m/s)	86 ± 32
Mitral E/A ratio	1.6 ± 1.3
Mitral E/e’_mean_	10 ± 5
TR peak velocity (m/s)	3.5 ± 0.8
RVSP (mmHg)	56 ± 22

NTproBNP, N-terminal pro-B-type natriuretic peptide; RAP, right atrial pressure; PAP, pulmonary artery pressure; PCWP, pulmonary capillary wedge pressure; TPG, transpulmonary gradient; PVR, pulmonary vascular resistance; LV, left ventricle; EF, ejection fraction; RV, right ventricular; TAPSE, tricuspid annular plane systolic excursion; TR, tricuspid regurgitation.

**Table 3:** Sensitivity, specificity, positive predictive value, negative predictive value for mitral E/A + age and ASE/EACVI algorithm to identify PCWP > 15 mmHg in the derivation cohort.

	Feasibility (%)	Sensitivity (%)	Specificity(%)	Positive predictive value (%)	Negative predictive value (%)	Accuracy (%)
Mitral E/A + Age	95	68	97	91	88	89
2016 ASE/EACVIalgorithm	87	75	93	32	89	87

### 3.3 Diagnostic accuracy of PVR_echo_

PVR_echo_ could be estimated in 88 of 111 patients (79%) employing PAMP_echo_ and PCWP_echo_ in the Ohm’s relationship. When compared with those in whom PVR_echo_ could not be assessed (n = 23; 21%), patients with quantifiable PVR_echo_ demonstrated higher PA pressures and PVR on RHC, and lower TAPSE on echocardiography (p < 0.05 for all comparisons).

**Fig. 5:**
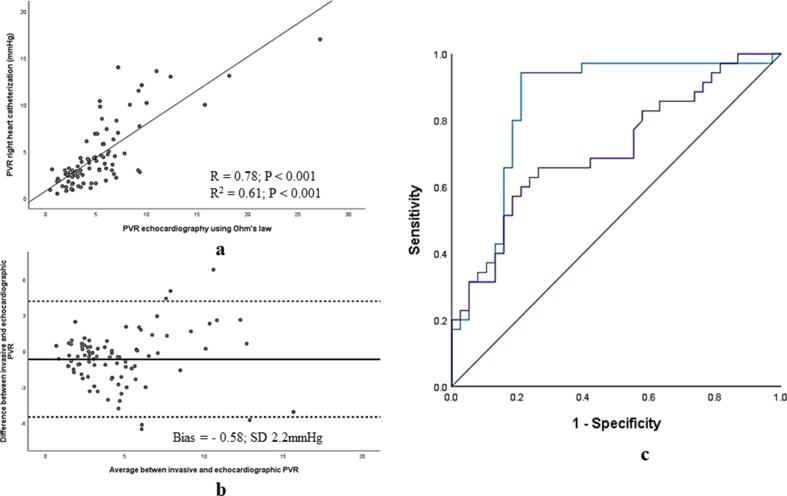
(a) Scatter plot displaying association between PVR_echo_ and PVR_RHC_ (b) Bland-Altman analysis demonstrating excellent agreement between PVR_echo_ andPVR_RHC_ in the derivation cohort and (c) comparision of diagnostic performance employing PVR_echo_ by Ohm’s relationship (AUC = 0.85) and Abbas algorithm (AUC = 0.70) in the derivation cohort (p = 0.02 for comparision).

PVR_echo_ demonstrated strong association (r = 0.78, r^2^ = 0.61; p < 0.001), negligible bias and excellent agreement with PVR_RHC_ on Bland-Altman analysis (Bias = − 0.58, SD 2.2 mmHg). **(**Fig. 5**a & 5b)** Further, this novel assessment of PVR outperformed conventional echocardiographic assessment using Abbas method (5) to identify elevated invasive PVR > 3WU (AUC = 0.85, CI 0.76 to 0.93 vs. AUC = 0.70, CI 0.58 to 0.81; p = 0.02 for comparison of AUC curves) **(**Fig. 5**c)**

**Fig. 6:**
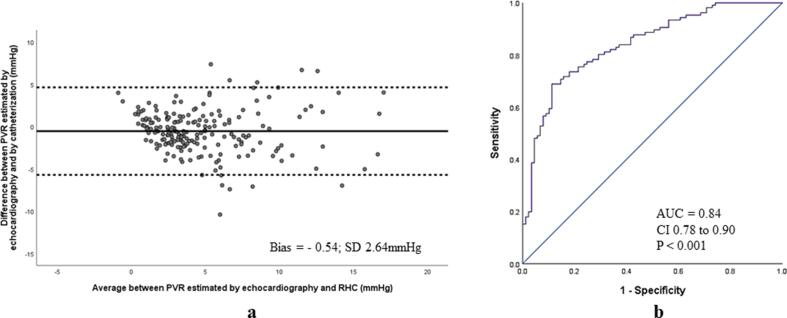
(a) Bland-Altman analysis demonstrating excellent agreement between PVR_echo_ and PVR_RHC_ in the validation cohort (b) diagnostic performance of PVR_echo_ to identify PVR_RHC_ > 3WU in the validation cohort.

### 3.4 External validation of PVR_echo_

We then validated the novel PVR_echo_ in an independent database of 238 symptomatic patients with normal sinus rhythm referred for clinically-indicated RHC to the PH referral center at the Karolinska University Hospital. Baseline characteristics of this cohort are presented in Table 2. This population demonstrated a higher proportion of patients with PH (n = 192; 81% vs. 68% in the Umeå cohort) and elevated PVR (61% vs. 48% respectively). Among those with PH, 121 (63%) demonstrated pre-capillary PH and 71(37%), post-capillary PH. Among post-capillary PH patients, 44 (62%) showed isolated post-capillary PH and 27 (38%) demonstrated combined post- and pre-capillary PH.

In the validation cohort, PVR_echo_ demonstrated minimal bias and excellent agreement with PVR_RHC_ (bias = -0.54; SD 2.64 mmHg) (Fig. 6a) and strong diagnostic ability to identify PVR_RHC_ > 3WU (AUC = 0.84, CI 0.78 to 0.90; p < 0.001) (Fig. 6b). PVR_echo_ > 3WU demonstrated 88% sensitivity, 54% specificity, 69% PPV and 79% NPV to identify PVR_RHC_ > 3WU (*Kappa* coefficient 0.43). A relatively higher agreement with RHC was obtained when PVR_echo_ > 4.6WU was employed as cut-off (*Kappa* coefficient 0.55; 72% sensitivity, 83% specificity, 84% PPV and 71% NPV). Further, PVR _echo_ was significantly higher among combined post- and pre-capillary PH patients when compared with isolated post-capillary PH (5.9 ± 3.7 vs 2.8 ± 2.7WU, p = 0.001) and demonstrated good diagnostic performance to discriminate these two groups (AUC = 0.77, CI 0.64 to 0.89; p = 0.001).

## 4 Discussion

We propose a novel echocardiographic approach to assess PVR employing variables routinely obtained in daily clinical practice using the hydraulic analogy to Ohm’s law. Simplified Doppler-based estimates of PCWP and PAMP employed in this equation demonstrated negligible bias and excellent agreement with corresponding invasive measurements. PVR_echo_ obtained using this approach was highly feasible, demonstrated strong diagnostic performance and outperformed traditional echocardiographic algorithms to assess PVR_RHC_. When validated in an independent hemodynamic database of patients referred for PH evaluation, PVR_echo_ preserved strong agreement with RHC measurements, showed excellent ability to identify elevated PVR_RHC_ and strong diagnostic capability to differentiate isolated post-capillary from combined post- and pre-capillary PH. Our findings showcase PVR_echo_ as a promising, non-invasive surrogate of reference-standard PVR that may be useful in diagnosis and regulating PH therapy.

### 4.1 Age-dependent mitral E/A ratio to represent PCWP

While the mitral E/A ratio is highly feasible and integral to the assessment of diastolic dysfunction, it demonstrates well-recognized limitations that prevent its use as an independent surrogate of elevated LV filling pressures as per current recommendations [16] First, the E/A ratio showcases a U-shaped relation with LV diastolic function. In the specific setting of normal LV function, both subjects with normal and elevated PCWP_RHC_ can demonstrate E/A ratio between 1 and 2. However, for values over 2, a sensitivity of 43% and specificity of 99% for identifying elevated PCWP_RHC_ has been reported. [17] Further, both age and gender are known to significantly affect mitral doppler indices of diastolic dysfunction and age has been earlier shown to be the strongest independent predictor of mitral E/A. [18] An observed shift from a normal transmitral filling pattern to an ‘abnormal’ relaxation pattern is not unusual with aging, suggesting that absolute cut-offs may not be suited to the diagnosis of diastolic dysfunction. More complex algorithms have been recently proposed to evaluate PCWP_RHC_. Recently, a model combining TR velocity. E/e’, LV EF, RV fractional area change, IVC diameter and LA volume demonstrated a sensitivity of 92%, specificity of 93% and area under the curve of 0.97 to estimate elevated PCWP_RHC_. [9] However, such an algorithm necessitates acquisition of several measures incorporating considerable inter- and intra-observer variability in the approach. Our data suggests that considering age in addition to mitral E/A (which demonstrated strongest correlation with PCWP_RHC_) offers a simple, pragmatic measure with strong diagnostic performance.

### 4.2 Echocardiographic evaluation of PAMP

In this study, PAMP_echo_ was assessed using the validated relationship proposed by Aduen et al. [19] and Chemla et al. [13] Assessment of pulmonary artery systolic pressure has traditionally been performed by adding an RAP estimate derived from IVC size and respiratory dynamics to the *trans*-tricuspid gradient.[12] Recent studies, however, suggest that these RAP estimates are frequently inaccurate and do not improve agreement with invasive reference. [14] Application of a fixed, representative value maintained strong association and minimal bias with invasive PA pressures in the aforementioned study. Our cohort demonstrated a limited spread of invasive RAP (Median 6 mmHg, IQR 4 to 10 mmHg) measurements and no significant differences when patients with elevated and normal PVR_RHC_ were compared. In this context, a fixed RAP estimate simplifies assessment of PAMP_echo_ using the Chemla approach [13], retains strong agreement with invasive measurements, and overcomes inherent technical limitations associated with IVC assessment.[20]

One can argue that the assessment of PVR_echo_ as employed in this study requires assessment of PAMP_echo_, PCWP_echo_ and CO_echo_, and each variable introduces a margin of error. However, we have chosen a pragmatic, simplified approach to assess highly reproducible variables routinely assessed in echocardiography labs worldwide. The variables chosen demonstrate higher feasibility and our approach demonstrates lower complexity when compared with more recently proposed models. [9] Advanced speckle-tracking has shown promise in estimation of RAP [21] and potentially improve estimation of PA pressures but this approach demonstrates relatively lower reproducibility and is rarely utilized in clinical practice.

### 4.3 Comparison with other Doppler-based PVR assessment

Our novel approach to assess PVR_echo_ outperformed the conventional Doppler-based algorithm postulated by Abbas and colleagues. [5] The Abbas algorithm was originally tested in a pre-capillary PH population with preserved EF, and one can speculate that this approach may generate false-positives and showcase lower accuracy in a population that includes HF patients with post-capillary PH. However, comparison with other echocardiographic methods to estimate PVR [6], [7], [8] needs to be explored in further studies. Another strength of the current approach is its reasonable ability to distinguish isolated post-capillary PH from combined post- and pre-capillary PH in the validation cohort, although this may need to be further investigated in larger populations.

Beyond PVR, the Ohm’s law relationship considering surrogates of pressure and flow has also been utilized to evaluate systemic vascular resistance in the setting of heart failure [22] and cardiogenic shock. [23] Novel non-invasive approaches such as these may be valuable in monitoring therapeutic interventions [24] and need to be further validated in larger databases.

### 4.4 Clinical implications

Accurate, non-invasive estimation of PVR employing commonly available echocardiographic variables taking age into consideration may improve patient screening and triaging for invasive catheterization in addition to regulating therapy during follow-up. In addition, this approach may be useful to distinguish PH hemodynamic subgroups where PVR evaluation determines therapeutic management.

### 4.5 Limitations

Although micromanometer-tipped catheters offer high-fidelity pressure recordings and are considered the invasive standard, we employed standard fluid-filled catheters that are routinely utilized in clinical practice. Analysis of echocardiographic images in the validation and derivation sites were performed by two experienced operators employing standard international recommendations, thereby minimizing inter-evaluator variability.

### 4.6 Conclusions

PVR_echo_ estimated employing the hydraulic analogy to Ohm’s Law is highly feasible, demonstrates excellent agreement with invasive measurements and identifies elevated PVR_echo_ with high accuracy. This novel, pragmatic approach to non-invasive PVR assessment may be of value in patient screening, diagnosis and PH therapy regulation.

## Declaration of Competing Interest

The authors declare that they have no known competing financial interests or personal relationships that could have appeared to influence the work reported in this paper.
